# MRI of ASCT2-mediated amino acid uptake in xenograft tumor models

**DOI:** 10.21203/rs.3.rs-7367339/v1

**Published:** 2025-10-26

**Authors:** Behnaz Ghaemi, Balaji Krishnamachary, Natalie Dillman, Kirsten N. Bains Williams, Yuki Hodo, Yuguo Li, Marie-France Penet Vidaver, Martin G. Pomper, Caitlin M. Tressler, Zaver M. Bhujwalla, Michael T. McMahon, Jeff W.M. Bulte, Peter C.M. Zijl, Aline M. Thomas

**Affiliations:** Johns Hopkins University; Johns Hopkins University; Johns Hopkins University; Johns Hopkins University; Johns Hopkins University; Kennedy Krieger Institute; Johns Hopkins University; University of Texas Southwestern Medical Center; Johns Hopkins University; Johns Hopkins University; Kennedy Krieger Institute; Johns Hopkins University; Johns Hopkins University; Johns Hopkins University

## Abstract

We investigated alanine as a magnetic resonance imaging (MRI) biomarker for alanine-serine-cysteine transporter 2 (ASCT2), the primary transporter for glutamine in cancer. Alanine exhibited higher contrast using the chemical exchange saturation transfer (CEST) method than other major ASCT2 substrates. Upon bolus injection of alanine (6 mmole/kg), CEST MRI enhancement *in vivo* was higher in *SLC1A5*-overexpressing Pa20c pancreatic tumors than naïve tumors (p < 0.0001). Enhancement was more pronounced in LNCaP prostate tumors than DU-145 prostate tumors *in vivo* (p < 0.0001). The temporal dynamics of alanine-weighted CEST (ALAwCEST) signal enhancement depended on the volume of the tumor, which histological analysis revealed to be due to alterations in the expression and distribution of ASCT2 and CD31-positive blood vessels. Mass spectrometric imaging of ^13^C-labeled metabolites confirmed differences in alanine uptake and metabolism in prostate tumors. We demonstrated the feasibility of ALAwCEST imaging for reporting ASCT2-mediated uptake in multiple human cancer models.

## Introduction

Cellular glutamine dependence is an attractive target for cancer imaging and therapy. Glutamine is actively transported into the cell, upon which it is metabolized to serve as a nitrogen and carbon source for biosynthesis of amino acids and nucleosides and as a supplemental energy source^1^. Glutamine transporters have been bi-directionally linked to the activation of oncogenes: Mdm2^2^, which has been shown to enhance the expression of these transporters, and Myc^3^, which is activated by them. Alanine-serine-cysteine transporter 2 (ASCT2; gene: *SLC1A5*), the primary transporter for glutamine import, is associated with poor prognosis for many cancers^4–8^ and their therapies^9^. At the clinical stage, several glutamine antagonists that include ASCT2 inhibition as their mode of action have been tested, though none beyond phase I trials^10^. In preclinical stages, multiple inhibitors have been developed to target this transporter^11,12^. Assays for measuring the transport capabilities of ASCT2 *ex vivo* are not yet commercially available.

Histopathological grading of its expression is a current clinical metric for ASCT2. For prostate cancer, higher ASCT2 expression corresponds with cancer recurrence^9^. Still, expression level is not proportional to cell uptake by ASCT2, unless glycosylated to translocate from the cytoplasm to the plasma membrane^13^. In addition, functionality of ASCT2 is sodium-dependent and alterations in the transporters responsible for regulating sodium levels have been reported in multiple cancers^14^. If a suitable MRI-detectable substrate could be found, *in vivo* MRI may allow evaluation of ASCT2-mediated transport and report on the tumor environment.

We hypothesized that natural substrates could serve as chemical exchange saturation transfer (CEST) magnetic resonance imaging (MRI) agents for evaluating ASCT2 activity. CEST MRI has the advantage of detecting low-concentration molecules (mM level) without modification or the need for specialized equipment through the presence of water-exchanging protons, for instance in their amine groups^15^. In this report we investigated the potential utility of several natural amine-containing substrates as CEST imaging biomarkers for ASCT2 activity, namely: alanine, asparagine, glutamine, serine, and threonine. Next, we measured CEST contrast upon intravenous injection of alanine in mice bearing Pa20c pancreatic tumors engineered to express different levels of ASCT2. Afterwards, we evaluated alanine-weighted CEST (ALAwCEST) contrast *in vivo* for DU-145 and LNCaP prostate tumors that differentially express ASCT2. Subsequently, we correlated ALAwCEST enhancement to ASCT2 expression in these tumors. Lastly, we correlated ALAwCEST enhancement to the uptake and metabolism of ^13^C-labeled alanine using matrix-assisted laser desorption/ionization (MALDI) mass spectrometry imaging.

## Results

### CEST MRI of Major ASCT2 Substrates

**Figure 1:**
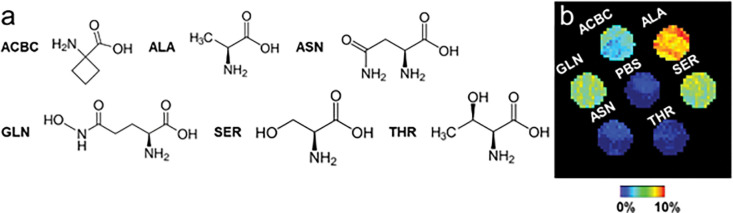
CEST imaging ASCT2 substrates. (**A**) Chemical structure and (**B**) CEST signal at +3.1 ppm and 37 °C of major, natural ASCT2 substrates compared to the synthetic ASCT2 substrate ACBC (from which fluciclovine is derived). Phantoms consisted of 20 mM solutions of these metabolites in PBS titrated to a pH of 7.3. ACBC = 1-aminocyclobutane carboxylate. Ala = alanine. Asn = asparagine. Gln = glutamine. Ser = serine. Thr = threonine.

**Figure 2:**
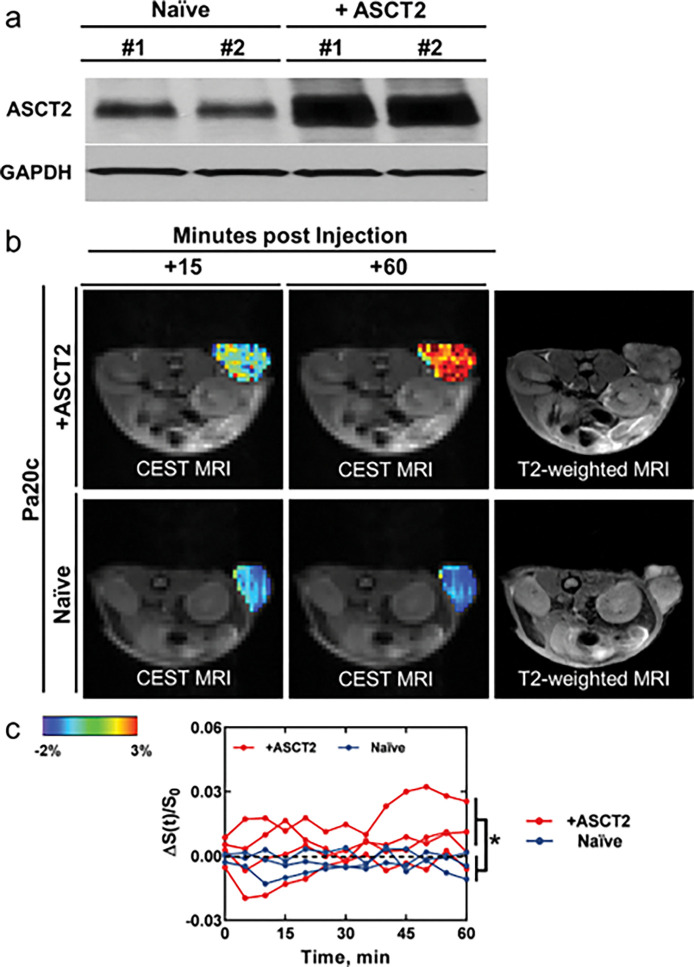
Dynamic CEST enhancement in genetically-engineered pancreatic tumors upon alanine injection. (A) Western blot of ASCT2 expression levels in naïve and genetically-engineered Pa20c tumors. (B) Visualization of CEST enhancement at the +3.1 ppm frequency over the course of the scan upon administration of alanine. (C) Quantification of ALAwCEST enhancement over the course of the scan in naïve and ASCT2-overexpressing Pa20c tumors. * = p < 0.05. n = 4 for the +ASCT2 group and n = 3 for the naïve group.

Initially, we assessed the potential of naturally occurring CEST MRI agents to report on ASCT2 activity. We screened 5 natural amino acids that are major substrates of ASCT2—glutamine, alanine, serine, threonine, and asparagine^16^—in PBS solution (20 mM, pH of 7.3, T = 37°C) for amineCEST signal generation (Fig. 1). They were also compared to ACBC (1-aminocyclobutane carboxylic acid), the nonfluorinated synthetic amino acid from which [18F]-fluciclovine ([18F]-FACBC) is derived. Notably, glutamine^17^, asparagine^17^ and ACBC^18^ can also be transported by the LAT1 amino acid exchanger. Of them, alanine generated the highest signal, followed by glutamine and serine, and then ACBC. Threonine and asparagine generated minimal signal compared to PBS alone. Thus, we continued investigating alanine based on its high CEST signal, as well as its relatively good clinical safety profile^19^ compared to the other ASCT2 substrates. Next, we evaluated the sensitivity of ALAwCEST MRI to ASCT2 activity using a genetically-engineered cancer model. We characterized ASCT2 expression in naïve and *SLC1A5*-overexpressing Pa20c patient-derived pancreatic xenograft tumors (Fig. 2) and confirmed that *SLC1A5*-overexpression in this cell line increased ASCT2 protein levels in its tumors. Then, we compared their CEST signal enhancement at the + 3.1 ppm frequency upon intravenous alanine injection (6 mmol/kg). As shown in Fig. 2c, CEST signal remained close to baseline levels over a period of 60 min following infusion in naïve Pa20c tumors (n = 3), but amine-weighted CEST (i.e. ALAwCEST) rose steadily in Pa20c tumors engineered to express higher levels of ASCT2 (n = 4). Their CEST enhancement significantly differed (p < 0.0001).

### Alanine-Weighted CEST MRI of Prostate Tumor Models

Subsequently, we assessed the potential of ALAwCEST to differentiate naïve prostate tumor models with different levels of ASCT2 expression and glutamine uptake. We characterized ASCT2 expression (Fig. S1) in prostatic cancer cell lines DU-145 and LNCaP using fluorescent microscopy. The average intensity of ASCT2 in LNCaP was 11% higher than that of DU-145 (p = 0.0203). Since cellular uptake by ASCT2 requires translocation of the transporter to the plasma membrane, we investigated the intensity across the cell area. A line profile revealed expression at the cellular border was higher than that in the cytoplasm for LNCaP cells. It also revealed that the cell line had higher fluorescent intensity compared to DU-145 across the cellular space. Since ALAwCEST signal could be dependent on downstream enzymatic conversion, we also quantified the expression of alanine transaminase (ALT2) and glutamate dehydrogenase 1 (GDH1) in these cells (Fig. S2). The average intensity of ALT2 and GDH1 was 58% (p = 0.0078) and 25% (p = 0.0190) higher in LNCaP than DU-145, respectively. Furthermore, since hypoxia is a common feature of prostate tumors, we also evaluated changes in their expression after short-term (24 h) exposure to hypoxia (1%). Within that time frame we found ASCT2 expression was significantly lower (5%: p = 0.0108) in LNCaP cells exposed to hypoxic conditions compared to LNCaP cells exposed to normoxic conditions. We then characterized changes in the CEST signal of their tumor spheres (100 μL cell volume) when exposed to alanine (400 μL of 10 mM; Fig. S3). CEST signal in LNCaP-containing phantoms was higher than DU-145-containing phantoms (p = 0.0090) and the alanine only control phantoms (p = 0.0021) after 4 hours of exposure (210 minutes after the initial acquisition). ALAwCEST enhancement of DU-145 containing phantoms was also higher than alanine only control phantoms (p = 0.0012).

**Figure 3:**
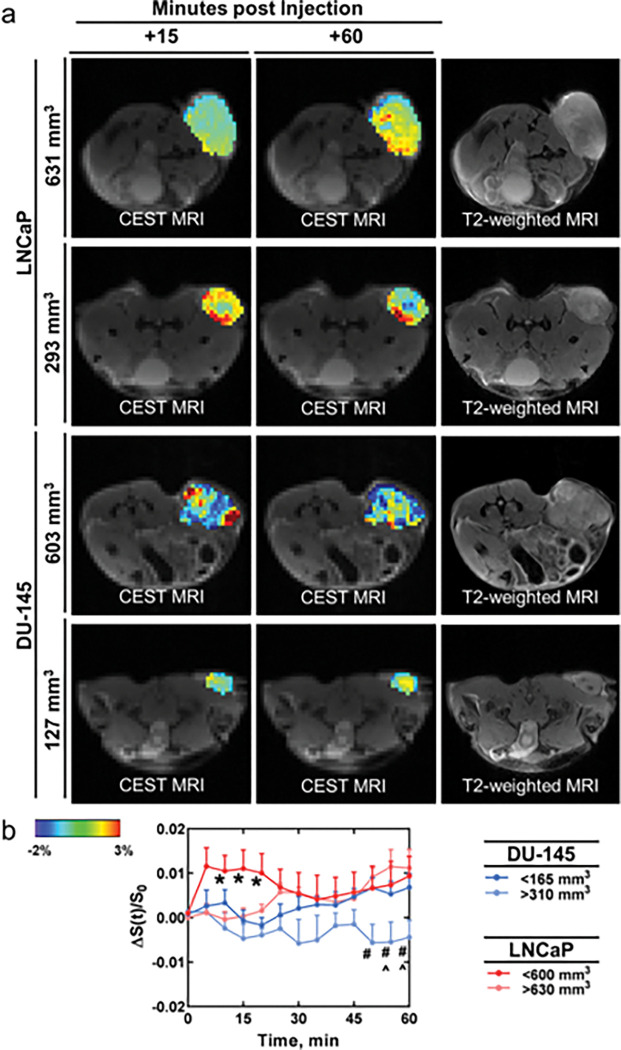
Dynamic CEST enhancement in prostate tumors upon alanine injection. (**A**) Visualization of CEST enhancement at the +3.1 ppm frequency over the course of the scan. (**B**) Quantification of CEST enhancement over the course of the scan in LNCaP tumors and DU-145 tumors. * = p < 0.05 of larger DU-145 tumors (>310 mm^3^) compared to the smaller LNCaP tumors (< 600mm^3^). # = p < 0.05 of larger LNCaP (>630 mm^3^) compared to the larger DU-14 5 (> 310 mm^3^) tumors. ^ = p < 0.05 of smaller LNCaP (<600 mm^3^) compared to the larger DU-14 5 (> 310 mm^3^) tumors. n = 5 and n = 3 for LNCaP tumors that were < 600 mm^3^ and > 630 mm^3^, respectively. n = 5 and n = 4 for DU-145 tumors that were < 165 mm^3^ and > 310 mm^3^, respectively. Individual enhancement curves are in supplemental figures 4 and 5.

Subsequently, we compared ALAwCEST signal enhancement between LNCaP (n = 8) and DU-145 (n = 9) tumors. We observed necrotic cores in some of the larger DU-145 tumors (> 630 mm^3^, similar to others^20^) and excluded those mice from further analysis. In dynamic CEST scans, CEST enhancement was higher in LNCaP tumors than DU-145 tumors (p < 0.0001). In static CEST scans, acquired before and after the dynamic ones, CEST contrast at the + 3.0 to + 3.2 ppm frequency range and the − 3.2 to −3.0 ppm frequency range did not significantly change upon administration of alanine. We then stratified tumors by volume. ALAwCEST enhancement profiles differed visually in LNCaP tumors with volumes less than 600 mm^3^ (n = 5) and greater than 630 mm^3^ (n = 3) when alanine was administered (Fig. S4). For smaller tumors (< 600 mm^3^), CEST signal reached a peak approximately 15 minutes after agent injection, returned to baseline approximately 45 minutes after agent injection, and then plateaued. For larger tumors (> 630 mm^3^), CEST signal steadily increased during the scan session. CEST signal dynamics between these two size classes significantly differed (p = 0.04). CEST enhancement profiles differed visually in DU-145 tumors with volumes less than 165 mm^3^ (n = 5) and greater than 310 mm^3^ (n = 4) when alanine was administered (Fig. S5). ALAwCEST signal at the + 3.1 ppm frequency was higher than baseline for smaller tumors (< 165 mm^3^), but lower than baseline for larger tumors (> 310 mm^3^). Their CEST signal dynamics significantly differed (p < 0.0001). CEST enhancement at the end of the dynamic scan negatively correlated (ρ = −0.7579, p = 0.02) with tumor volume. CEST signal enhancement amongst the volume-stratified tumor groups were then compared (Fig. 3). At early times (~ 15 minutes after alanine injection), signal at the + 3.1 ppm frequency was significantly (0.008 < p < 0.043) higher in smaller LNCaP tumors (< 600 mm^3^) compared to larger DU-145 tumors (> 310 mm^3^). Towards the end of the scan session, both smaller (< 600 mm^3^: 0.0261 < p < 0.0427) and larger (> 630 mm^3^: 0.0148 < p < 0.0497) LNCaP tumor groups had significantly higher CEST enhancement than larger DU-145 tumors (> 310 mm^3^).

### Fluorescent Imaging of ASCT2 Content in Prostate Tumors

**Figure 4:**
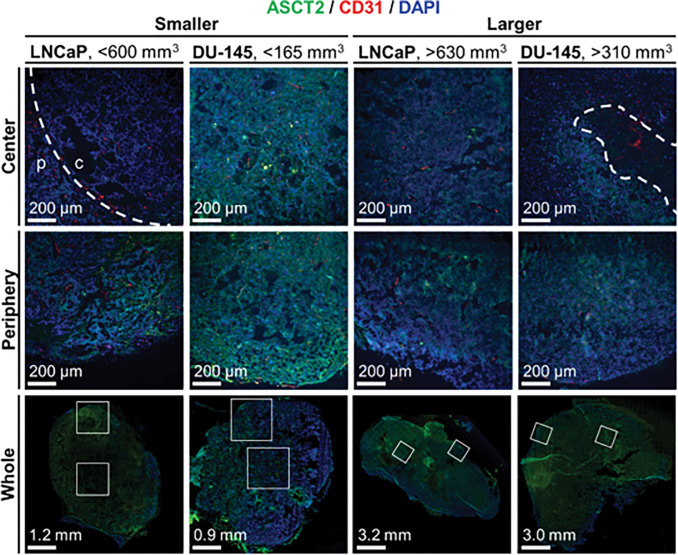
Expression of ASCT2 and CD31 in prostate tumors. Visualization of ASCT2 (green) and CD31 (red) expression in (left) smaller and (right) larger DU-145 and LNCaP at the (top) center region or (bottom) periphery of the tumor. DAPI staining (blue) of nucleic structures is shown for reference. White squares outline the location of the magnified images above.

Next, we characterized ASCT2 and CD31 expression in larger and smaller LNCaP and DU-145 tumors (Fig. 4, Fig. S6). Fluorescent intensity of ASCT2 was highest in smaller DU-145 tumors. Levels were comparable in the periphery of smaller LNCaP tumors, but were much lower in the center, reminiscent of the ring of bright CEST signal observed in Fig. 3. ASCT2 staining intensity in the periphery of larger DU-145 tumors was comparable to that in larger LNCaP tumors, but in the center of larger DU-145 tumors, staining levels were low and heterogenous proximal to necrotic (acellular) lesions. Endothelial (CD31) levels were generally comparable, in line with what was reported in the literature for these two tumors^21–24^. Still, CD31 staining was lower in the center of smaller LNCaP and larger DU-145 tumors. In the former, CD31 staining patterns were similar to ASCT2 staining patterns, i.e. ring-like. In the latter, CD31 staining was also heterogenous, but localized independently of ASCT2 staining.

### MALDI Mass Spectrometry Imaging of Prostate Tumor Uptake and Metabolism of Alanine

**Figure 5:**
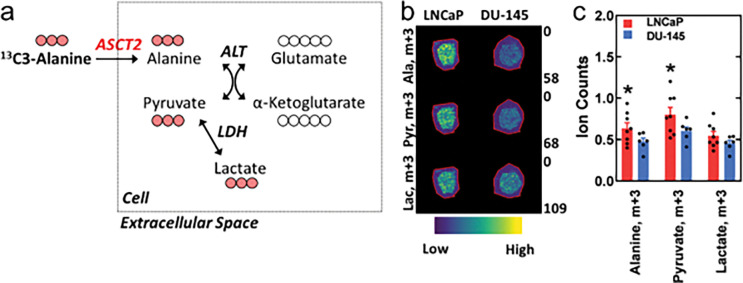
MALDI imaging of ^13^C3-alanine and its downstream metabolic products. (A) Schematic of the carbon (pink) flow from the imaging agent to its metabolic products prior to tricarboxylic acid (TCA) cycling. (B) Visualization and (C) quantification of ^13^C-labeled metabolites in LNCaP and DU-145 tumors. Metabolites: Ala=alanine, Lac=lactate, and Pyr=pyruvate. Proteins: αKGDH=alpha-ketoglutarate dehydrogenase, ALT=alanine transaminase, ASCT2=alanine serine cysteine transporter 2, GDH=glutamate dehydrogenase, GLS=glutaminase, GS=glutamine synthetase, IDH=isocitrate dehydrogenase, and LDH=lactate dehydrogenase. * = p < 0.05. Mean ± s.e.m. n = 8 for the LNCaP group and n = 6 for the DU-145 group

To demonstrate that the source of the CEST MRI signal enhancement was the uptake of alanine, we compared the presence of ^13^C3-labeled alanine and its metabolites in tumors *ex vivo* using isotopomer analysis of MALDI mass spectrometry images (Fig. 5). The presence of ^13^C3-alanine (p = 0.0329) and its immediately downstream metabolic product ^13^C3-pyruvate (generated intracellularly by alanine transaminase: p = 0.0357) was higher in LNCaP tumors than in DU-145 tumors. In contrast, the presence of ^13^C3-lactate, a product of the CEST agent entering the glycolytic pathway, was similar (p = 0.0877) amongst the tumors.

## Discussion

Although best known for transporting glutamine, the neutral amino acid transporter ASCT2 has multiple, naturally-occurring substrates^16,25–27^, i.e. alanine, asparagine, glycine, leucine, methionine, serine, threonine, and valine. All of these substrates can be metabolized via their respective transaminases^28^. We investigated the utility of CEST MRI to detect and image ASCT2-mediated transport using some of these unmodified, naturally occurring substrates. We then selected alanine from this pool of candidate agents in part due to its readiness for translation. Alanine is a well-recognized substrate for ASCT2 that has been injected intravenously at a dose of 6 mmole/kg^29^ into humans for other applications without symptoms. Its suitability as an ASCT2 agent for cancer imaging has already been demonstrated with PET imaging in preclinical models using a fluorinated version of the metabolite^30^. In addition, ^13^C-labeled alanine^31^ has also been injected in humans and is readily hyperpolarized^32^, permitting spectroscopic imaging. Most recently, its T2 relaxation was leveraged to permit imaging of a glioblastoma model (U87MG) using MRI^33^. The relatively low toxicity of alanine in humans permitted us to use high doses for intravenous administration in our experiments, which should be translatable to the clinic. Its relatively high safety profile^19^ has the added advantage of enabling the repetition of these scans, which is beneficial for monitoring therapeutic efficacy in cancer management. We elected to investigate alanine-mediated CEST enhancement in multiple tumor xenograft models. The use of genetically-engineered pancreatic tumor models enabled us to establish the feasibility of alanine to report ASCT2 activity. The use of the DU-145 and LNCaP prostate cancer models enabled us to establish the sensitivity. Their relative expression (protein) of ASCT2 is well-established^9,34,35^ and the uptake of ASCT2 substrates in LNCaP cells is reported to be nearly 1.5 times faster than that of DU-145 cells^36,37^. Still, compared to other cancers, prostate cancer expression (mRNA) is quite low, ranking fourth from last according to a systematic review^38^. This combination of attributes permitted us to examine the utility of dynamic ALAwCEST MRI at the lower limits of ASCT2 for cancer applications.

When naturally occurring molecules are used, CEST enhancement is dependent on not only the pH and concentration of the agents in tissue compartments, but also on the concentration of their metabolized products when the latter contain the same exchangeable proton groups^39–44^. In the tumor microenvironment intracellular pH becomes more alkaline, reducing CEST contrast for amine groups. On the other hand, pH in the interstitium (extracellular, extravascular space) acidifies, increasing amine proton contrast^45–49^. Reported values of intracellular and extracellular pH in prostate cancer models range from 7.1 to 7.6^50–52^ and from 6.4 to 6.8^53–55^, respectively. In larger (> 310 mm^3^) DU-145 tumors the directionality of CEST enhancement was slightly negative (Fig. S5), which could result from the lower ASCT2 levels observed in histological images compared to the smaller tumors (Fig. 4, Fig. S6). Since hypoxia is often featured in larger^56–58^, more advanced^59^ tumors, particularly prostate cancer^60^, we investigated its impact on their expression in DU-145 and LNCaP cells. We did not observe significant alterations in ASCT2 expression (protein) in DU-145 cells when cultured in hypoxic conditions *in vitro*, but the same conditions decreased ASCT2 expression in LNCaP cells (Fig. S2) and increased ASCT2 expression (mRNA, protein) in pancreatic cancer cell lines^61^. Still, decreased ASCT2 expression and/or function may occur under longer-term exposure. Alternatively, low glutamine availability—which is associated with less vascularized tumors such as the larger DU-145 tumors in this report (Fig. 4)—has been shown to decrease ASCT2 expression in liver^62^ and gastric^63^ cancer cell lines. In larger LNCaP tumors (cutoff between 600–630 mm^3^), a decreased vascular fraction also explains the size-dependence of CEST profiles for LNCaP tumors as the initial peak seen in smaller tumors was not present in larger tumors. Peaks within the first 30 minutes has been observed with other CEST agents delivered at these doses^41^. Additionally, early-stage blood vessel marker CD31 revealed larger LNCaP tumors were less vascularized than smaller ones (Fig. 4, Fig. S6).

MALDI mass spectrometry imaging was used in our studies to validate the uptake of our CEST agents (Fig. 5). MALDI imaging confirmed the presence of ^13^C-labeled alanine in tumor tissue. However, similar to *in vivo* imaging techniques, mass spectrometry imaging is unable to differentiate the compartment, i.e. vascular, extracellular and intracellular spaces, of these metabolites unless they are known not to be transportable. To minimize the contribution of the vascular and extracellular compartments to this measurement, we extracted the tumors after multiple half-lives (< 15 minutes^64,65^). Furthermore, as alanine is metabolized inside the cell, we compared differences in the presence of its ^13^C-labeled metabolic products to indirectly confirm the occurrence of cellular uptake. These findings warrant more extensive *in vivo* and *ex vivo* metabolic studies to establish the fate of this imaging agent and improve the interpretation of its images.

There are several limitations to our studies. Firstly, we did not image pH *in vivo* or *ex vivo* to evaluate its impact on our measurement of ASCT2 function. Still, several MRI and magnetic resonance spectroscopy methods to measure pH have been translated to human use^66^ that can be used to evaluate its contribution. Secondly, we did not image tumor vascular function to evaluate its impact on CEST enhancement and MALDI images. However, CD31 staining (Fig. S6) revealed vessel density in DU-145 and LNCaP tumors was comparable. Furthermore, the blood clearance half-lives of small molecules such as alanine in rodent blood are less than 15 minutes^64,65^; therefore, the contribution of the vascular fraction to the CEST signal would be expectedly minimal by the end of the scan session. Still, we recognize that the measurement and consideration of these confounding factors would improve the interpretation of ASCT2 imaging in the clinic. Gadolinium-enhanced MRI is already a feature of cancer patient care used to evaluate the vascular profile of tumors. These evaluations would complement the visual information provided by ALAwCEST enhancement.

In conclusion, we evaluated alanine for imaging ASCT2-mediated glutamine transport. ALAwCEST MRI detected its uptake without modification via the water-exchanging protons of its amine group. Dynamic CEST imaging of alanine was able to differentiate Pa20c-naïve and Pa20c-ASCT2 pancreatic tumor models as well as DU-145 and LNCaP prostate tumor models. The direction and magnitude of dynamic ALAwCEST enhancement when alanine was administered was sensitive to size-dependent differences in the ASCT2 content, particularly for DU-145 tumors. Lastly, as alanine has been intravenously injected into humans for other applications at a dose (6 mmole/kg) that should allow ALAwCEST MRI detection, ASCT2 imaging using this metabolite warrants further optimization and development as its use as a noninvasive, natural imaging agent is readily translatable.

## Methods

### Prostate cell line culture.

For monolayer cultures, the prostatic cancer lines DU-145 (ATCC, 1 × 10^4^) and LNCaP (ATCC, 2 × 10^5^) were seeded onto 6-well plates and cultured in RPMI media (Gibco, A10491–01) supplemented with fetal bovine serum (10%; Gibco, 25140–079) and penicillin-streptomycin (1%; Gibco, 15140–122). For spheroid culture, DU-145 cells (1.5 × 10^3^) and LNCaP (1.5 × 10^4^) cells were seeded onto low-attachment 6-well plates (Corning Costar^®^) and cultured in DMEM/F12 media (Gibco, 10565–018) supplemented with 1% penicillin-streptomycin, B27 (2%; Gibco 17504–044), insulin (3 μg/ml; Sigma, I2643) epidermal growth factor (20 ng/mL; Gibco, PHG0311), and knock-out serum (3%; Gibco, 10828–010) until a 100 μL volume of cells was attained.

### Pancreatic cell line generation and culture.

The Pa20c (Panc198/RRID: CVCL_E285, male) human pancreatic cancer cell line is from a primary pancreatic tumor characterized previously in a published work^67^ that was kindly provided by Dr. Anirban Maitra (MD Anderson Cancer Center). To generate ASCT2-overexpressing Pa20c cells, a 1.62 kb open reading frame (ORF) of human solute carrier family 1 member 5 (SLC1A5) transcript variant 1 (ASCT2) was spliced into a lentiviral vector (LV-231-SLC1A5-eGFP) purchased from GeneCopoeia (Cat. No. EX-A6442-Lv231, Rockville, MD) and selected using puromycin. Virions for expressing the ASCT2 gene were produced by co-transfecting 293T cells with 12 μg of lentiviral construct, 6 μg of packaging plasmid pCMVΔR8.2 DVPR (VPR deleted) and 1.5 μg of pCMV-VSVg plasmid using lipofectamine 2000 (Invitrogen, Carlsbad, CA). Supernatant containing the virions were collected 48 h after transfection and gently centrifuged to remove cell debris. Pa20c cells were transfected daily with fresh lentivirus in serum-free media with 1μL/mL of polybrene (Sigma-Aldrich) for 1 week. These cells were cultured in DMEM (Sigma-Aldrich) with 10% FBS, 25 mmol/L glucose, and 4 mmol/L glutamine.

### Immunocytochemistry.

Prostatic cancer cell lines were cultured in standard conditions until approximately 50% confluency. One set of each cell line was left to incubate 24 hours at 37°C in standard incubating conditions (20% oxygen). The other set was placed for the same amount of time in an incubator set at 1% oxygen, which was reported by multiple groups to induce changes in protein expression in these cell lines^68–70^. Afterwards, cells were fixed using paraformaldehyde (4%, Sigma) for 10 minutes, permeabilized with Triton-X (0.1%) for 30 minutes, stained overnight with rabbit anti-ASCT2 (Cell Signaling Technology, cat. no. 5345; 1:100), rabbit anti-ALT2 (ProteinTech, 16757–1-AP; 1:400), or goat anti-GDH1 (Invitrogen, PA5–19267; 1:400), and then stained for 1 hr using the following secondary antibodies: donkey anti-goat AF647 (1:200) or anti-rabbit AF488 (1:200) antibodies. Data shown is representative of 4–5 independent characterizations.

### Histology.

Tumors were fixed with paraformaldehyde (2%) via cardiac perfusion and frozen (−80°C) until use. Upon cryosectioning the tissues to 14 μm thick slices, they were permeabilized with Triton-X (0.1%) and stained overnight with rat anti-CD31 (Novus Biologicals, NB600–1475, 1:200) and with rabbit anti-ASCT2 (1:100). Donkey anti-rat AF488 and donkey anti-rabbit AF594 were used as secondary antibodies.

### Western blot.

Pa20c-naïve and Pa20c-ASCT2 cells were lysed for whole-cell protein extraction using RIPA (Radio Immuno Precipitation Assay) buffer (Sigma-Aldrich) following the manufacturer’s instructions. Protein (100 μg total) was resolved on a 4–15% gradient SDS-PAGE gel. Proteins were transferred to a nitrocellulose membrane and then incubated with the rabbit anti-ASCT2 antibody overnight. Mouse anti-GAPDH monoclonal antibody (Sigma, 1:5000) was used as loading control. After washing, the membrane was incubated with a horseradish peroxidase-conjugated donkey anti-rabbit or anti-mouse secondary antibody (GE healthcare, 1:2000) for 1 hour. Immunoblots were developed using SuperSignal^™^ West Pico PLUS Chemiluminescent Substrate kit (Thermo Fisher) following the manufacturer’s instructions.

### MALDI mass spectrometry imaging.

Tumors were embedded in M1 media (Thermo Fisher), cryosectioned at a 30 μm slice thickness and placed on indium tin oxide-coated glass slides (Delta Technologies). Tissue sections were warmed to room temperature (RT) in a vacuum desiccator for 10 mins prior to spraying. 1,5-diaminonaphthalene (10 mg/mL) in 70% acetonitrile with 0.1% trifluoroacetic acid was applied using an HTX M5 sprayer (HTX Technologies) with the following parameters: 30-degree nozzle temperature, 4 passes, 0.1 ml/min flow rate, 1200 mm/min velocity, 2.5 mm track spacing criss-cross spray pattern, 10 psi pressure, and 2 L/min gas flow rate. MALDI imaging was performed in reflectron-negative mode at a 100-micron pixel and raster size with 200 laser shots per pixel using a Bruker RapifleX MALDI TOF/TOF instrument.

### Metabolite phantom preparation and MRI.

Alanine (Sigma, PHR1110), 1-aminocyclobutane carboxylic acid (ACBC; Sigma, 652369), asparagine (Sigma, A4159), glutamine (Sigma, G3126), serine (Sigma, S4311), or threonine (Sigma, T8441) were dissolved separately in phosphate buffered saline (PBS). Solutions (20 mM) were titrated to a pH of 7.3 ± 0.03 and placed into 5 mm NMR tubes (Wilmad, WG-1000–7) for MR imaging at 37°C. Phantom images were acquired using a Bruker 9.4T vertical bore spectrometer and a 20 mm coil. Amine CEST maps were generated from MTR_asym_ spectra. Scan parameters were TR/TE = 8000/5.58 ms, RARE factor = 32, NA = 1, repetitions = 1, B_1_ = 3.6 μT, saturation length (block pulse) = 4 s, frequency sweep from − 8 to + 8 ppm with 0.1 ppm increments, matrix size = 76 × 76, slice thickness = 2 mm, and field of view = 20 × 20 mm^2^. Spectral shifts due to B_0_ inhomogeneity were corrected for using the water saturation shift referencing (WASSR) method^71,72^.

### Cell phantom preparation and MRI.

The cell-rich layer consisted of prostate cancer spheres placed on top of a layer of agarose (1% in PBS) in 5 mm NMR tubes at a height of 5 mm (~ 100 μL in volume), confirmed using a caliper. The supernatant-rich layer consisted of PBS (pH of ~ 7.3; 400 μL) with bovine serum albumin (BSA; 1%) and alanine (10 mM). Phantoms without cells that contained PBS with BSA with or without alanine served as controls. Phantom images of the cell-rich and supernatant-rich layers were acquired in alternation at 37°C using a Bruker 11.7T vertical bore spectrometer and a 20 mm coil. CEST enhancement maps were generated from Z-spectra. Scan parameters were TR/TE = 10000/4.53 ms, RARE factor = 32, NA = 1, repetitions = 1, B_1_ = 3.6 μT, saturation length = 3 s, frequency sweep from − 8 to + 8 ppm with 0.4 ppm increments, matrix size = 64 × 64, slice thickness = 2 mm, and field of view = 20 × 20 mm^2^. B_0_ inhomogeneity was corrected using the WASSR method^71^.

### Tumor induction and MRI.

Animal work performed was approved by the institutional ACUC. DU-145 (n = 9, 1–2 × 10^6^) or LNCaP (n = 8, 2–3 × 10^6^) prostatic cancer cells were subcutaneously injected into the flank of male, 6–10 week old Rag2 mice (Jackson Laboratory, #8449). Pa20c-naïve (n = 3, 1–3 × 10^6^) and Pa20c-ASCT2 (n = 4, 1–3 × 10^6^) pancreatic cancer cell lines were subcutaneously injected into the flank of male 6–10 week old NCI Scid NCr mice (Charles River, #561). Mice were imaged 1–2 months after tumor induction using a Bruker 11.7T horizontal bore spectrometer and an 8-element phased-array surface coil. Animals were anesthetized with isoflurane (1–2%). Before and after dynamic CEST imaging, static CEST imaging was performed using a frequency sweep from − 8 to + 8 ppm with 0.2 ppm increments with a scan time of 14 minutes. Dynamic CEST images were collected at the + 3.1 ppm amine proton frequency prior to alanine injection (6 mmol/kg in bolus) to establish a baseline and over 60 minutes after agent injection. Dynamic CEST enhancement ΔS(t)/S_0_ was quantified by subtracting signal S/S_0_ at time t from the average signal obtained prior to injection. S_0_ (signal at + 40 ppm) was collected at the beginning and end of the scan (n = 5 each) and then linearly fitted over time to correct for drift. Static ALAwCEST enhancement was quantified from Z-spectra (ΔS/S_0_) and from MTR_asym_ spectra (ΔΔS/S_0_) by subtracting the signal in the ± 3.0 to ± 3.2 ppm range after injection from the signal before injection. CEST MRI scan parameters for *in vivo* images were TR/TE = 10000/3.49 ms, RARE factor = 32, NA = 1, repetitions = 1, B_1_ = 3.6 μT, saturation length = 3 s, matrix size = 48 × 48, slice thickness = 1.5 mm, and field of view = 30 × 30 mm^2^. For static CEST maps and spectra, B_0_ inhomogeneity was corrected using the WASSR method as before^71^. Tumor size (mm^3^) was calculated as the sum of the cross-sectional areas of T2-weighted images multiplied by the slice thickness.

### Statistics.

Student’s t-tests, paired or unpaired, and two-way ANOVAs were performed as appropriate. Spearman’s rank was used for correlations. Significance was defined as p < 0.05. Histograms of the intensity frequencies were normalized (by pixel number) and averaged. Line profiles are from representative regions as outlined in the figure.

## Supplementary Material

Supplementary Files

This is a list of supplementary files associated with this preprint. Click to download.

• ASCT2SI.docx

## Data Availability

The datasets used and/or analysed during the current study are available from the corresponding author on reasonable request.

## References

[1] Choi Y. K. & Park K. G. Targeting Glutamine Metabolism for Cancer Treatment. Biomol Ther (Seoul) 26, 19–28 (2018). 10.4062/biomolther.2017.178 29212303 PMC5746034

[2] Riscal R. Chromatin-Bound MDM2 Regulates Serine Metabolism and Redox Homeostasis Independently of p53. Molecular Cell 62, 890–902 (2016). 10.1016/j.molcel.2016.04.033 27264869

[3] Yue M., Jiang J., Gao P., Liu H. & Qing G. Oncogenic MYC Activates a Feedforward Regulatory Loop Promoting Essential Amino Acid Metabolism and Tumorigenesis. Cell Reports 21, 3819–3832 (2017). 10.1016/j.celrep.2017.12.002 29281830

[4] Roux C. Endogenous glutamine decrease is associated with pancreatic cancer progression. Oncotarget 8 (2017). 10.18632/oncotarget.20545 PMC5707027 29221133

[5] Sun H.-W. GLUT1 and ASCT2 as Predictors for Prognosis of Hepatocellular Carcinoma. PLOS ONE 11, e0168907 (2016). 10.1371/journal.pone.0168907 28036362 PMC5201247

[6] Shimizu K. ASC amino-acid transporter 2 (ASCT2) as a novel prognostic marker in non-small cell lung cancer. British Journal of Cancer 110, 2030–2039 (2014). 10.1038/bjc.2014.88 24603303 PMC3992511

[7] Luo Y. ASCT2 overexpression is associated with poor survival of OSCC patients and ASCT2 knockdown inhibited growth of glutamine-addicted OSCC cells. Cancer Medicine 9, 3489–3499 (2020). 10.1002/cam4.2965 32162845 PMC7221297

[8] Bernhardt S. Proteomic profiling of breast cancer metabolism identifies SHMT2 and ASCT2 as prognostic factors. Breast Cancer Research 19, 112 (2017). 10.1186/s13058-017-0905-7 29020998 PMC5637318

[9] Wang Q. Targeting ASCT2-mediated glutamine uptake blocks prostate cancer growth and tumour development. The Journal of Pathology 236, 278–289 (2015). 10.1002/path.4518 25693838 PMC4973854

[10] Shen Y.-A. Inhibition of glutaminolysis in combination with other therapies to improve cancer treatment. Current Opinion in Chemical Biology 62, 64–81 (2021). 10.1016/j.cbpa.2021.01.006 33721588 PMC8570367

[11] Choi Y.-K. & Park K.-G. Targeting Glutamine Metabolism for Cancer Treatment. Biomolecules & Therapeutics 26, 19–28 (2018). 10.4062/biomolther.2017.178 29212303 PMC5746034

[12] Feng Y. Identification and Characterization of IMD-0354 as a Glutamine Carrier Protein Inhibitor in Melanoma. Molecular Cancer Therapeutics 20, 816–832 (2021). 10.1158/1535-7163.Mct-20-0354 33632871 PMC8102370

[13] Console L., Scalise M., Tarmakova Z., Coe I. R. & Indiveri C. N-linked Glycosylation of human SLC1A5 (ASCT2) transporter is critical for trafficking to membrane. Biochimica et Biophysica Acta (BBA) - Molecular Cell Research 1853, 1636–1645 (2015). 10.1016/j.bbamcr.2015.03.017 25862406

[14] Leslie T. K. Sodium homeostasis in the tumour microenvironment. Biochimica et Biophysica Acta (BBA) - Reviews on Cancer 1872, 188304 (2019). 10.1016/j.bbcan.2019.07.001 31348974 PMC7115894

[15] van Zijl P. C. M. & Yadav N. N. Chemical exchange saturation transfer (CEST): What is in a name and what isn’t? Magnetic Resonance in Medicine 65, 927–948 (2011). 10.1002/mrm.22761 21337419 PMC3148076

[16] Utsunomiya-Tate N., Endou H. & Kanai Y. Cloning and Functional Characterization of a System ASC-like Na+-dependent Neutral Amino Acid Transporter*. Journal of Biological Chemistry 271, 14883–14890 (1996). 10.1074/jbc.271.25.14883 8662767

[17] Yanagida O. Human L-type amino acid transporter 1 (LAT1): characterization of function and expression in tumor cell lines. Biochimica et Biophysica Acta (BBA) - Biomembranes 1514, 291–302 (2001). 10.1016/S0005-2736(01)00384-4 11557028

[18] Goodman M. M., Yu W. & Jarkas N. Synthesis and biological properties of radiohalogenated α,α-disubstituted amino acids for PET and SPECT imaging of amino acid transporters (AATs). Journal of Labelled Compounds and Radiopharmaceuticals 61, 272–290 (2018). 10.1002/jlcr.3584 29143354

[19] Garlick P. J. The Nature of Human Hazards Associated with Excessive Intake of Amino Acids. The Journal of Nutrition 134, 1633S–1639S (2004). 10.1093/jn/134.6.1633S 15173443

[20] Tan Z. Saturation transfer properties of tumour xenografts derived from prostate cancer cell lines 22Rv1 and DU145. Scientific Reports 10, 21315 (2020). 10.1038/s41598-020-78353-8 33277574 PMC7718243

[21] Mussawy H. The bone microenvironment promotes tumor growth and tissue perfusion compared with striated muscle in a preclinical model of prostate cancer in vivo. BMC Cancer 18, 979 (2018). 10.1186/s12885-018-4905-5 30326868 PMC6192198

[22] Virus Adeno-Associated 2-Mediated Intratumoral Prostate Cancer Gene Therapy: Long-Term Maspin Expression Efficiently Suppresses Tumor Growth. Human Gene Therapy 16, 699–710 (2005). 10.1089/hum.2005.16.699 15960601

[23] Li Y., Zhong W., Zhu M., Li M. & Yang Z. miR-185 inhibits prostate cancer angiogenesis induced by the nodal/ALK4 pathway. BMC Urology 20, 49 (2020). 10.1186/s12894-020-00617-2 32366240 PMC7197131

[24] Kai L. Targeting prostate cancer angiogenesis through metastasis-associated protein 1 (MTA1). The Prostate 71, 268–280 (2011). 10.1002/pros.21240 20717904

[25] Scopelliti A. J., Font J., Vandenberg R. J., Boudker O. & Ryan R. M. Structural characterisation reveals insights into substrate recognition by the glutamine transporter ASCT2/SLC1A5. Nature Communications 9, 38 (2018). 10.1038/s41467-017-02444-w PMC5750217 29295993

[26] Yao D. A Novel System A Isoform Mediating Na^+^/Neutral Amino Acid Cotransport *. Journal of Biological Chemistry 275, 22790–22797 (2000). 10.1074/jbc.M002965200 10811809

[27] Foster A. C. D-Serine Is a Substrate for Neutral Amino Acid Transporters ASCT1/SLC1A4 and ASCT2/SLC1A5, and Is Transported by Both Subtypes in Rat Hippocampal Astrocyte Cultures. PLOS ONE 11, e0156551 (2016). 10.1371/journal.pone.0156551 27272177 PMC4896441

[28] Vettore L., Westbrook R. L. & Tennant D. A. New aspects of amino acid metabolism in cancer. British Journal of Cancer 122, 150–156 (2020). 10.1038/s41416-019-0620-5 31819187 PMC7052246

[29] Fernandes J. & Blom W. The intravenous L-alanine tolerance test as a means for investigating gluconeogenesis. Metabolism - Clinical and Experimental 23, 1149–1156 (1974). 10.1016/0026-0495(74)90031-6 4372509

[30] Liu H. 18F-Alanine Derivative Serves as an ASCT2 Marker for Cancer Imaging. Molecular Pharmaceutics 15, 947–954 (2018). 10.1021/acs.molpharmaceut.7b00884 29308900

[31] Battezzati A., Haisch M., Brillon D. J. & Matthews D. E. Splanchnic utilization of enteral alanine in humans. Metabolism - Clinical and Experimental 48, 915–921 (1999). 10.1016/S0026-0495(99)90229-9 10421236

[32] Ji X. Transportable hyperpolarized metabolites. Nature Communications 8, 13975 (2017). 10.1038/ncomms13975 PMC5234073 28072398

[33] Yang S.-H. MRI measurement of alanine uptake in a mouse xenograft model of U-87 MG glioblastoma. Magnetic Resonance Imaging 93, 189–194 (2022). 10.1016/j.mri.2022.08.015 36029935

[34] Cardoso H. J. Glutaminolysis is a metabolic route essential for survival and growth of prostate cancer cells and a target of 5α-dihydrotestosterone regulation. Cellular Oncology 44, 385–403 (2021). 10.1007/s13402-020-00575-9 33464483 PMC12980676

[35] Hodo Y. Imaging the uptake and metabolism of glutamine in prostate tumor models using CEST MRI. npj Imaging 3, 34 (2025). 10.1038/s44303-025-00100-3 40750673 PMC12316899

[36] Okudaira H. Accumulation of Trans-1-Amino-3-[18F]Fluorocyclobutanecarboxylic Acid in Prostate Cancer due to Androgen-Induced Expression of Amino Acid Transporters. Molecular Imaging and Biology 16, 756–764 (2014). 10.1007/s11307-014-0756-x 24943499 PMC5648589

[37] Oka S. Transport mechanisms of trans-1-amino-3-fluoro[1–14C]cyclobutanecarboxylic acid in prostate cancer cells. Nuclear Medicine and Biology 39, 109–119 (2012). 10.1016/j.nucmedbio.2011.06.008 21958853

[38] Liu Y. The role of ASCT2 in cancer: A review. European Journal of Pharmacology 837, 81–87 (2018). 10.1016/j.ejphar.2018.07.007 30025811

[39] Rivlin M. & Navon G. CEST MRI of 3-O-methyl-D-glucose on different breast cancer models. Magnetic Resonance in Medicine 79, 1061–1069 (2018). 10.1002/mrm.26752 28497566

[40] Anemone A. In vitro and in vivo comparison of MRI chemical exchange saturation transfer (CEST) properties between native glucose and 3-O-Methyl-D-glucose in a murine tumor model. NMR in Biomedicine 34, e4602 (2021). 10.1002/nbm.4602 34423470 PMC9285575

[41] Rivlin M., Horev J., Tsarfaty I. & Navon G. Molecular imaging of tumors and metastases using chemical exchange saturation transfer (CEST) MRI. Scientific Reports 3, 3045 (2013). 10.1038/srep03045 24157711 PMC7365327

[42] Walker-Samuel S. In vivo imaging of glucose uptake and metabolism in tumors. Nature Medicine 19, 1067–1072 (2013). 10.1038/nm.3252 PMC5275770 23832090

[43] Nasrallah F. A., Pagès G., Kuchel P. W., Golay X. & Chuang K.-H. Imaging Brain Deoxyglucose Uptake and Metabolism by Glucocest MRI. Journal of Cerebral Blood Flow & Metabolism 33, 1270–1278 (2013). 10.1038/jcbfm.2013.79 23673434 PMC3734779

[44] Knutsson L., Xu X., van Zijl P. C. M. & Chan K. W. Y. Imaging of sugar-based contrast agents using their hydroxyl proton exchange properties. NMR in Biomedicine n/a, e4784 10.1002/nbm.4784 PMC9719573 35665547

[45] Ellingson B. M. pH-weighted molecular MRI in human traumatic brain injury (TBI) using amine proton chemical exchange saturation transfer echoplanar imaging (CEST EPI). NeuroImage: Clinical 22, 101736 (2019). 10.1016/j.nicl.2019.101736 30826686 PMC6396390

[46] Harris R. J. pH-weighted molecular imaging of gliomas using amine chemical exchange saturation transfer MRI. Neuro-Oncology 17, 1514–1524 (2015). 10.1093/neuonc/nov106 26113557 PMC4648305

[47] Wermter F. C., Bock C. & Dreher W. Investigating GluCEST and its specificity for pH mapping at low temperatures. NMR in Biomedicine 28, 1507–1517 (2015). 10.1002/nbm.3416 26412088

[48] Yao J., Wang C. & Ellingson B. M. Influence of phosphate concentration on amine, amide, and hydroxyl CEST contrast. Magnetic Resonance in Medicine 85, 1062–1078 (2021). 10.1002/mrm.28481 32936483 PMC8258865

[49] Cho N. S. Amine-weighted chemical exchange saturation transfer magnetic resonance imaging in brain tumors. NMR in Biomedicine 36, e4785 (2023). 10.1002/nbm.4785 35704275 PMC13030910

[50] Lee Z.-W. Intracellular Hyper-Acidification Potentiated by Hydrogen Sulfide Mediates Invasive and Therapy Resistant Cancer Cell Death. Frontiers in Pharmacology 8 (2017). 10.3389/fphar.2017.00763 PMC5671507 29163155

[51] Wilson L. T. A new class of ratiometric small molecule intracellular pH sensors for Raman microscopy. Analyst 145, 5289–5298 (2020). 10.1039/D0AN00865F 32672252

[52] Furuya Y., Lundmo P., Short A. D., Gill D. L. & Isaacs J. T. The role of calcium, pH, and cell proliferation in the programmed (apoptotic) death of androgen-independent prostatic cancer cells induced by thapsigargin. Cancer Res 54, 6167–6175 (1994). 7954463

[53] Vāvere, A. L. A Novel Technology for the Imaging of Acidic Prostate Tumors by Positron Emission Tomography. Cancer Research 69, 4510–4516 (2009). 10.1158/0008-5472.Can-08-3781 19417132 PMC2690701

[54] Ibrahim-Hashim A. Systemic Buffers Inhibit Carcinogenesis in TRAMP Mice. Journal of Urology 188, 624–631 (2012). 10.1016/j.juro.2012.03.113 22704445 PMC3694604

[55] Korenchan D. E. Hyperpolarized in vivo pH imaging reveals grade-dependent acidification in prostate cancer. Oncotarget 10 (2019). 10.18632/oncotarget.27225 PMC6817439 31692908

[56] Dunst J. Tumor Volume and Tumor Hypoxia in Head andNeck Cancers. Strahlentherapie und Onkologie 179, 521–526 (2003). 10.1007/s00066-003-1066-4 14509950

[57] Gaustad J.-V., Simonsen T. G., Andersen L. M. K. & Rofstad E. K. Vascular abnormalities and development of hypoxia in microscopic melanoma xenografts. Journal of Translational Medicine 15, 241 (2017). 10.1186/s12967-017-1347-9 29183378 PMC5706333

[58] Kis A. *In Vivo* Imaging of Hypoxia and Neoangiogenesis in Experimental Syngeneic Hepatocellular Carcinoma Tumor Model Using Positron Emission Tomography. BioMed Research International 2020, 4952372 (2020). 10.1155/2020/4952372 32832549 PMC7428931

[59] Kiraga Ł. Changes in hypoxia level of CT26 tumors during various stages of development and comparing different methods of hypoxia determination. PLOS ONE 13, e0206706 (2018). 10.1371/journal.pone.0206706 30412628 PMC6226158

[60] Vaupel P., Kallinowski F. & Okunieff P. Blood Flow, Oxygen and Nutrient Supply, and Metabolic Microenvironment of Human Tumors: A Review1. Cancer Research 49, 6449–6465 (1989). 2684393

[61] Yoo H. C. A Variant of SLC1A5 Is a Mitochondrial Glutamine Transporter for Metabolic Reprogramming in Cancer Cells. Cell Metabolism 31, 267–283.e212 (2020). 10.1016/j.cmet.2019.11.020 31866442

[62] BUNGARD Claire I. & McGIVAN John D. Glutamine availability up-regulates expression of the amino acid transporter protein ASCT2 in HepG2 cells and stimulates the ASCT2 promoter. Biochemical Journal 382, 27–32 (2004). 10.1042/bj20040487 15175006 PMC1133911

[63] Ma H. Inhibition of Glutamine Uptake Improves the Efficacy of Cetuximab on Gastric Cancer. Integrative Cancer Therapies 20, 15347354211045349 (2021). 10.1177/15347354211045349 34590499 PMC8488517

[64] Kee A. J., Smith R. C., Gross A. S., Madsen D. C. & Rowe B. The effect of dipeptide structure on dipeptide and amino acid clearance in rats. Metabolism 43, 1373–1378 (1994). 10.1016/0026-0495(94)90030-2 7968592

[65] Qi Z. Serial determination of glomerular filtration rate in conscious mice using FITC-inulin clearance. American Journal of Physiology-Renal Physiology 286, F590–F596 (2004). 10.1152/ajprenal.00324.2003 14600035

[66] Kim H., Krishnamurthy L. C. & Sun P. Z. Brain pH Imaging and its Applications. Neuroscience 474, 51–62 (2021). 10.1016/j.neuroscience.2021.01.026 33493621

[67] Jones S. Core Signaling Pathways in Human Pancreatic Cancers Revealed by Global Genomic Analyses. Science 321, 1801–1806 (2008). 10.1126/science.1164368 18772397 PMC2848990

[68] Geng H. Interplay between hypoxia and androgen controls a metabolic switch conferring resistance to androgen/AR-targeted therapy. Nature Communications 9, 4972 (2018). 10.1038/s41467-018-07411-7 PMC6255907 30478344

[69] Ravenna L. Distinct Phenotypes of Human Prostate Cancer Cells Associate with Different Adaptation to Hypoxia and Pro-Inflammatory Gene Expression. PLOS ONE 9, e96250 (2014). 10.1371/journal.pone.0096250 24801981 PMC4011733

[70] Ma Y. Prostate Cancer Cell Lines under Hypoxia Exhibit Greater Stem-Like Properties. PLOS ONE 6, e29170 (2011). 10.1371/journal.pone.0029170 22216200 PMC3247249

[71] Thomas A. M., Xu J., Calabresi P. A., van Zijl P. C. M. & Bulte J. W. M. Monitoring diffuse injury during disease progression in experimental autoimmune encephalomyelitis with on resonance variable delay multiple pulse (onVDMP) CEST MRI. NeuroImage 204, 116245 (2020). 10.1016/j.neuroimage.2019.116245 31605825 PMC7767058

[72] Kim M., Gillen J., Landman B. A., Zhou J. & van Zijl P. C. M. Water saturation shift referencing (WASSR) for chemical exchange saturation transfer (CEST) experiments. Magnetic Resonance in Medicine 61, 1441–1450 (2009). 10.1002/mrm.21873 19358232 PMC2860191

